# Cone Dystrophy in Patient with Homozygous *RP1L1* Mutation

**DOI:** 10.1155/2015/545243

**Published:** 2015-01-29

**Authors:** Sachiko Kikuchi, Shuhei Kameya, Kiyoko Gocho, Said El Shamieh, Keiichiro Akeo, Yuko Sugawara, Kunihiko Yamaki, Christina Zeitz, Isabelle Audo, Hiroshi Takahashi

**Affiliations:** ^1^Department of Ophthalmology, Nippon Medical School, Chiba Hokuso Hospital, Inzai, Chiba 270-1694, Japan; ^2^INSERM, U968, 75012 Paris, France; ^3^Sorbonne Universités, UPMC Université Paris 06, UMR_S 968, Institut de la Vision, 75012 Paris, France; ^4^CNRS, UMR_7210, 75012 Paris, France; ^5^Honjo Daiichi Hospital, Yurihonjo, Akita 015-0834, Japan; ^6^Centre Hospitalier National d'Ophtalmologie des Quinze-Vingts, DHU ViewMaintain, INSERM-DHOS CIC 1423, 75012 Paris, France; ^7^Department of Ophthalmology, Nippon Medical School, Bunkyo-ku, Tokyo 113-8602, Japan

## Abstract

The purpose of this study was to determine whether an autosomal recessive cone dystrophy was caused by a homozygous *RP1L1* mutation. A family including one subject affected with cone dystrophy and four unaffected members without evidence of consanguinity underwent detailed ophthalmic evaluations. The ellipsoid and interdigitation zones on the spectral-domain optical coherence tomography images were disorganized in the proband. The proband had a reduced amplitude of cone and flicker full-field electroretinograms (ERGs). Focal macular ERGs and multifocal ERGs were severely reduced in the proband. A homozygous *RP1L1* mutation (c.3628T>C, p.S1210P) was identified in the proband. Family members who were heterozygous for the p.S1210P mutation had normal visual acuity and normal results of clinical evaluations. To investigate other putative pathogenic variant(s), a next-generation sequencing (NGS) approach was applied to the proband. NGS identified missense changes in the heterozygous state of the *PCDH15*, *RPGRIP1*, and *GPR98* genes. None of these variants cosegregated with the phenotype and were predicted to be benign reinforcing the putative pathogenicity of the *RP1L1* homozygous mutation. The AO images showed a severe reduction of the cone density in the proband. Our findings indicate that a homozygous p.S1210P exchange in the *RP1L1* gene can cause cone dystrophy.

## 1. Introduction

Mutations in the* retinitis pigmentosa 1-like 1 *(*RP1L1*) gene cause autosomal dominant occult macular dystrophy (OMD; OMIM 613587) [1–6]. The* RP1L1* gene was originally identified through sequence analyses of human and mouse genomes [7, 8]. The human* RP1L1 *gene has 4 exons that span 50 kb on chromosome 8p. The length of the mRNA of* RP1L1* is over 7 kb, but its exact length varies among individuals because of several different length polymorphisms.* RP1L1* encodes a protein with a maximal length of 2,480 amino acids and a predicted molecular weight of 252 kDa. The expression of RP1L1 protein is limited to the retina, and it appears to be specific to photoreceptors [7].* RP1L1* was found to be conserved in distant vertebrates [8]. Knockout mice that lack the RP1L1 protein have reduced electroretinograms (ERGs) and progressive photoreceptor degeneration [9]. Studies of* Rp1l1*−/− mice showed that the RP1L1 protein is located in the axoneme of the outer segments and connecting cilia of the photoreceptors [9].

Occult macular dystrophy (OMD; OMIM 613587) is an inherited macular dystrophy characterized by a progressive decrease in the visual acuity with an essentially normal fundus and normal fluorescein angiograms [10, 11]. The full-field electroretinograms (ERGs) are normal; however, the focal macular ERGs and multifocal ERGs (mfERGs) recorded from the macular area are reduced [10–12]. Spectral-domain optical coherence tomography (SD-OCT) showed various degrees of disruption of the ellipsoids and interdigitation zones in OMD patients [13, 14]. The cone and rod sensitivity profiles of OMD patients indicate depressed cone sensitivity in the macula, although many patients have normal rod sensitivity in the macula [15]. In many OMD patients, the waveform of the focal macular ERGs has a depolarizing pattern [2, 15]. The good preservation of rod function in the macula has been suggested to be related to this ERG finding [2, 15].

The adaptive optics (AO) fundus camera can obtain images with a transverse resolution of <2 *μ*m, which makes it possible to resolve individual cone photoreceptors and other structures in living human eyes [16–18]. This technique has been used to analyze the cone photoreceptor mosaic in eyes with inherited retinal degenerations [17, 19–21]. An increase in the cone spacing, that is, a reduction of cone density, in retinas with cone-rod dystrophy can be detected by AO imaging [17, 19, 20]. A dark area in the AO fundus images was reported to be caused by disruptions of the interdigitation zone in the SD-OCT images [22, 23].

The purpose of this study was to determine whether autosomal recessive cone dystrophy was caused by homozygous* RP1L1* mutations. To accomplish this, we performed detailed molecular genetic analysis including next-generation sequencing (NGS) on a patient with cone dystrophy and applied the NGS results to her family members. In addition, we performed high-resolution imaging of the retinal morphology with an adaptive optics (AO) fundus camera.

## 2. Patients and Methods

The protocol conformed to the tenets of the Declaration of Helsinki, and it was approved by the Institutional Review Board of the Nippon Medical School. A written informed consent was obtained from all patients after an explanation of the nature and possible consequences of the study.

### 2.1. Clinical Studies

The ophthalmological examinations included measurements of the best-corrected visual acuity (BCVA), refraction, slit-lamp biomicroscopy, ophthalmoscopy, fundus photography, perimetry, SD-OCT, fluorescein angiography (FA), full-field ERGs, focal macular ERGs, and mfERGs. The visual fields were determined by the Goldman perimetry or the Humphrey Visual Field Analyzer (Model 745i; Carl Zeiss Meditec, Inc, Dublin, California). The Swedish interactive threshold algorithm standard strategy was used with program 30-2 of the Humphrey Visual Field Analyzer. The OCT images were acquired with a Cirrus HD-OCT (Carl Zeiss Meditec).

### 2.2. Electroretinograms

Full-field scotopic and photopic ERGs were recorded using the extended testing protocol incorporating the International Society for Clinical Electrophysiology of Vision standards (LE2000, Tomey, Nagoya, Japan) [24]. Focal macular ERGs were recorded with a commercial Focal Macular ERG system (ER80; Kowa Company, Tokyo, Japan, and PuREC; Mayo Company, Nagoya, Japan) using a bipolar contact lens electrode (MY type Electrode; Mayo Company, Nagoya, Japan). The stimulus and background lights were integrated into an infrared fundus camera [25, 26]. The size of the stimulus spot was 15° in diameter and was placed on the macula by observing the infrared image of the retina on a monitor. The white stimulus and background illuminations were generated by light-emitting diodes that had maximal spectral emissions at 440 to 460 nm and 550 to 580 nm, respectively. The luminance of the stimuli was 115.7 cd/m^2^ and the background was 8.0 cd/m^2^. The duration of the stimuli was 100 milliseconds. The responses were amplified and filtered by digital band pass filters from 5 to 200 Hz. Three hundred responses were summed with a stimulus frequency of 5 Hz.

The mfERGs were recorded using a commercial mfERG system (VERIS Science; Electro-Diagnostic Imaging, Inc. Redwood City, CA, USA) [27, 28]. The mean luminance of stimulus was 103 cd/m^2^ and the contrast was 95%. The overall stimulus area subtended approximately 40 degrees diameter of visual angle, and the frame rate was 75 Hz. The pseudorandom stimulus presentation, the m-sequence, was 2^14^ − 1, and each run was divided into eight equal segments with a total recording time of about 4 min.

### 2.3. Mutation Analyses and Computational Assessments of Missense Mutations

**Figure 1:**
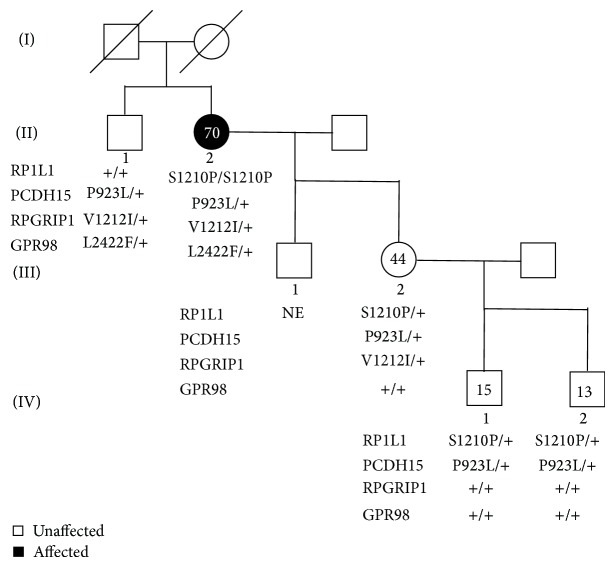
Pedigree of a family with cone dystrophy. Affected patient is shown with a solid symbol and unaffected with open symbols. Numbers in the symbols are ages of the family member when they were clinically examined. Sequencing results of the variants of* RP1L1, PCDH15, RPGRIP1,* and* GPR98* are shown below the symbols. Squares, male; circles, female; slashed symbols, deceased; +, wild type; NE, not examined.

Blood samples were collected from the proband (II-2) and her family members (II-1, III-2, IV-1, and IV-2; Figure 1), and genomic DNA was isolated from the peripheral white blood cells using a blood DNA isolation kit (NucleoSpin Blood XL; Macherey Nagel, Germany). The DNA was used as the template to amplify the* RP1L1* gene. The coding regions and flanking introns of the* RP1L1* gene were amplified by polymerase chain reaction (PCR) using published primers [2] synthesized by Greiner Bio-One (Tokyo, JAPAN). The PCR products were purified (ExoSAP-IT; USB Corp., USA) and were used as the template for sequencing. Both strands were sequenced on an automated sequencer (Bio Matrix Research; Chiba, JAPAN). The identified mutations were assayed in 460 control chromosomes from 230 healthy Japanese individuals by direct sequencing. The effect of a missense mutation of an encoded protein was predicted by PolyPhen-2, SIFT, PMut, and Align GVGD online tools [29–33].

### 2.4. Molecular Genetic Analysis Using Next-Generation Sequencing (NGS)

Targeted NGS analysis was performed according to methods described earlier [34] and subsequently revised and improved. Briefly, a custom-made SureSelect oligonucleotide probe library was designed to assess the exons of 123 genes implicated in different retinal disorders according to Agilent's recommendations (Table 1).

### 2.5. High-Resolution Imaging Analyses

High-resolution fundus images were obtained with an infrared AO retinal camera with a transverse resolution of 2.4 *μ*m (rtx1, Imagine Eyes, Orsay, France) [35]. This system has been used to image individual cone photoreceptors [21, 36–39] and other retinal structures [18, 36, 40]. Successive AO images are taken at adjacent retinal locations with an angular spacing of 2 degrees in the horizontal and vertical directions. This procedure allows an overlap of horizontal and vertical images of at least 2 degrees. We had acquired AO images of the photoreceptor mosaic at the depth of maximum cone image intensity. We had acquired AO images around 6 degrees to both nasal and temporal sides horizontally from the fovea. The resulting images were stitched together by superimposing retinal vessel landmarks with an image editing software (GIMP, The GIMP Development Team; Image J, National Institute of Health, Bethesda, MD). The size of each pixel was typically 0.8 *μ*m when calculated at the retinal plane, and the values were adjusted for variations in the axial length of the eye [41]. To evaluate the cone patterns of normal controls and Case III-2, we used the automated cone labeling analysis software (AOdetect; Imagine Eyes). AOdetect was developed by Imagine Eyes. The positions of the photoreceptors are computed by automatically detecting the central coordinates of small circular spots where the brightness is higher than the surrounding background level. First, the averaged image, without contrast adjustment, was filtered so that the local maxima of the image were detected. The spatial distribution of these points was analyzed using Voronoi diagrams where the detected points served as generators. After automated cone labeling, the estimated cone labeling was manually verified by three investigators to minimize any potential cone under- or oversampling made by the automated software. As has been reported for similar systems, we could clearly distinguish individual cones at >500 *μ*m from the fovea. Therefore, we obtained an estimate of cone density in a 100 × 100 *μ*m area at 600 *μ*m from the foveal center. We examined the cone density in 26 normal control eyes. There were 19 men and 7 women whose age ranged from 23 to 67 years (mean, 42 ± 12.7 years) in this control group. We calculated the 95% confidence intervals, 95% prediction intervals, and *R* ^2^ value of regression line of the cone density of normal controls. We have evaluated the statistical analysis at one eccentricity from the fovea.

The automated cone labeling did not estimate each cone precisely in the images taken from the region with severe photoreceptor degeneration. To estimate the cone density of Case II-2 in a 100 × 100 *μ*m area at 600 *μ*m from the foveal center, we manually selected circular spots more than 4 *μ*m in the images where the brightness was obviously higher than the surrounding background level. The density of the cones was manually measured by three investigators to minimize any potential cone under- or oversampling.

## 3. Results

### 3.1. Case Report

A 64-year-old woman (II-2) reported a gradual decrease of vision, and our examination showed that her decimal BCVA was 0.4 in the right eye and 0.3 in the left eye without obvious cataracts and fundus abnormalities. Family history revealed no other members with any eye disease including her parents who were deceased (Figure 1). She was referred for brain MRI to rule out cortical or optic nerve abnormalities because she had no obvious ocular abnormalities despite her decreased BCVA. The MRI findings were normal.

**Figure 2:**
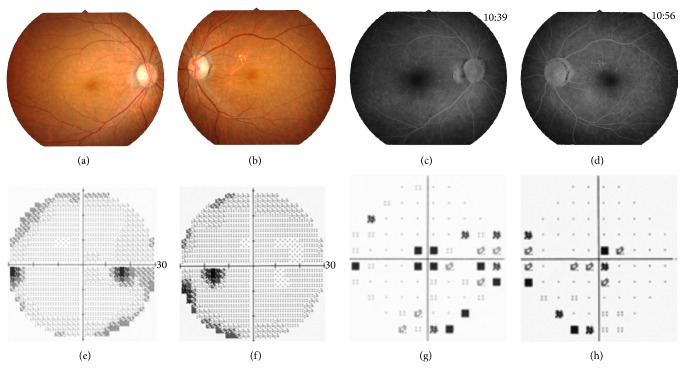
Clinical findings of the proband, Patient II-2. Fundus photographs (a, b) and fluorescein angiograms (c, d) show no abnormal findings. The results of the Humphrey Visual Field Analyzer show a relative reduction of the central sensitivity in both eyes (e–h).

**Figure 3:**
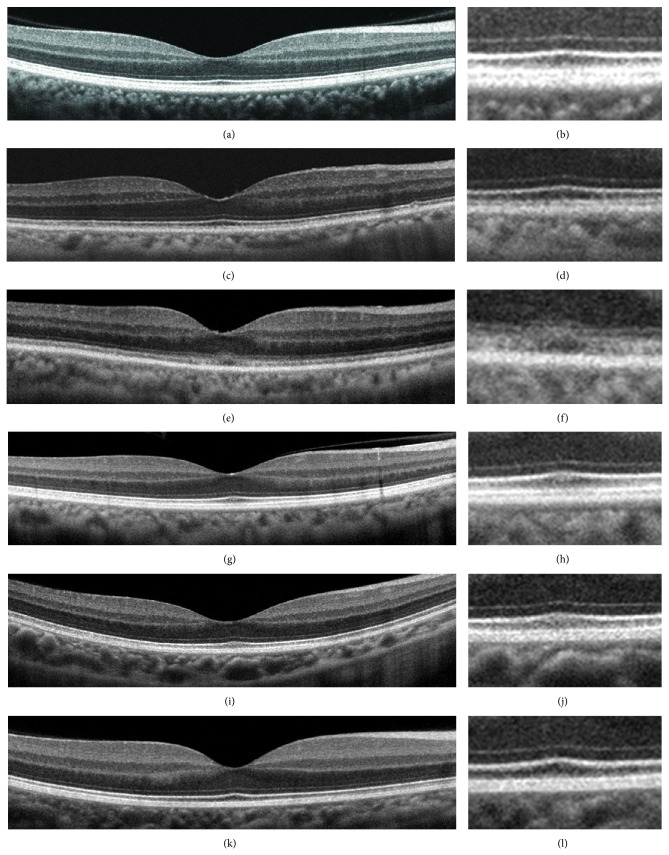
Spectral-domain optical coherence tomographic (SD-OCT) images. Images of normal control (a, b) and of family members (c–l). Images at lower magnification (a, c, e, g, i, and k) and higher magnification (b, d, f, h, j, and l) are shown. SD-OCT images from the right eye of a normal control (a, b), Case II-1 (c, d), Case II-2 (e, f), Case III-2 (g, h), Case IV-1 (i, j), and Case IV-2 (k, l) are shown. The SD-OCT findings of the eyes in Case II-2 show blurred ellipsoid and discontinuous interdigitation zones (e, f). Family member of the case showed clearly distinguishable ellipsoid and interdigitation zones in the central macular area.

**Figure 4:**
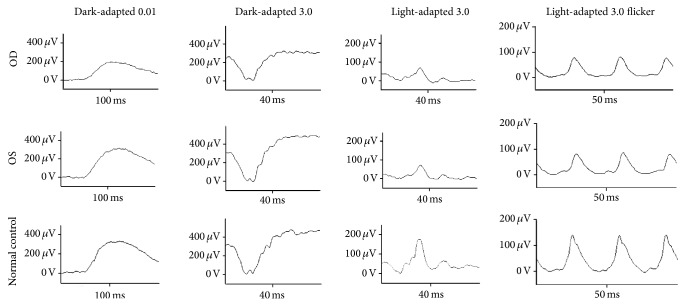
Full-field electroretinograms (ERGs). Full-field ERGs recorded from Case II-2 are shown. The dark-adapted 0.01, dark-adapted 3.0, light-adapted 3.0, and light-adapted 3.0 flicker ERGs are shown. The results of dark-adapted 0.01 and dark-adapted 3.0 ERGs show a slight reduction of the b-wave amplitudes of the right eye although the amplitudes are within normal limit of our institutional age-matched standard. The amplitudes of the b-wave of light-adapted 3.0 and light-adapted 3.0 flicker responses are markedly reduced in both eyes.

**Figure 5:**
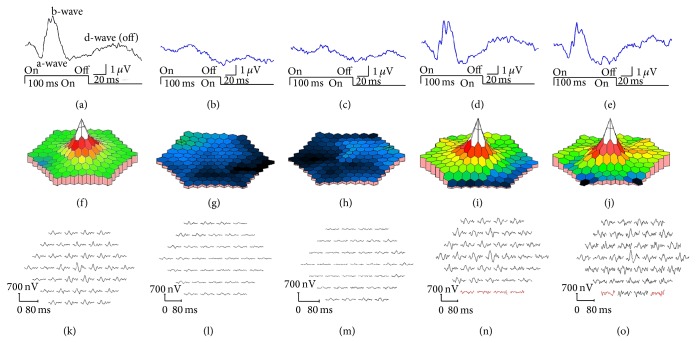
Results of focal macular ERGs and multifocal ERGs. Focal macular ERGs recorded from normal control (a), Case II-2 (b, c), and Case III-2 (d, e) are shown. The amplitudes of the a-wave and b-wave of Case II-2 are severely reduced. Topographic map (f–j) and local responses (k–o) of multifocal ERGs recorded from normal control (f, k), Case II-2 (g, h, l, and m), and Case III-2 (i, j, n, and o) are shown. The amplitudes of the foveal area are severely reduced in Case II-2. The results from right eyes (b, d, g, i, l, and n) and left eyes (a, c, e, f, h, j, k, m, and o) are shown. The amplitudes of the focal macular ERGs and mfERGs in proband's daughter (III-2) were within normal limits (Figures 5(d), 5(e), 5(i), 5(j), 5(n), and 5(o)).

Six years later, she was referred to our hospital to undergo cataract surgery. Her BCVAs were 0.3 in the right eye and 0.2 in the left eye. Slit-lamp examinations showed that both lenses had mild cortical opacities. Fundus examinations were normal (Figures 2(a) and 2(b)). A month later, cataract surgery was performed on both eyes without complications, but the visual acuity of both eyes did not improve. The findings of fluorescein angiography were normal (Figures 2(c) and 2(d)). The visual fields were full by Goldman perimetry, and a relative reduction of the central sensitivity was detected in both eyes by the Humphrey Visual Field Analyzer (Figures 2(e)–2(h)). The SD-OCT images showed blurred ellipsoid and discontinuous interdigitation zones at the fovea (Figures 3(e) and 3(f)). The a- and b-wave amplitudes of dark-adapted 0.01 and 3.0 full-field ERGs were mildly reduced in the right eye, but the amplitudes of both eyes were within the normal limits of our institutional age-matched controls (Figure 4). The amplitudes of the b-wave of the cone ERGs and the amplitude of the flicker responses were markedly reduced in both eyes (Figure 4). The amplitudes of the a- and b-waves of the focal macular ERGs were severely reduced (Figures 5(b) and 5(c)), and the amplitudes of the mfERGs in the central area were also severely reduced (Figures 5(g), 5(h), 5(l), and 5(m)).

### 3.2. Molecular Genetic Findings

**Figure 6:**
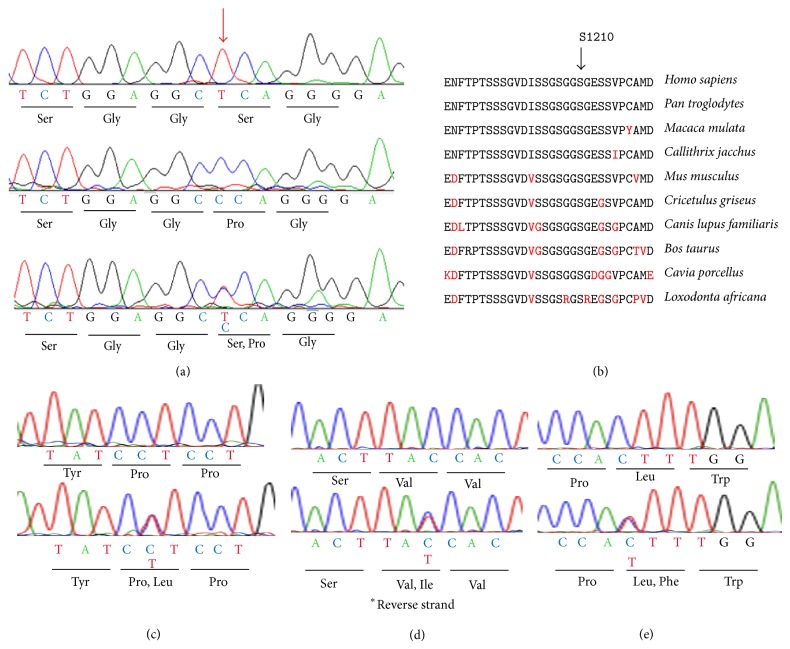
Molecular genetic results. (a) Sequence chromatograms for normal control (top), Case II-2 (middle), and Case III-2 (bottom) are shown. Case II-2 had a homozygous c.3628T>C mutation in exon 4. Case III-2 had a heterozygous c.3628T>C mutation. Position c.3628 is indicated by red arrow. (b) Alignment of amino acid position 1191 to 1220 of RP1L1 family proteins. Amino acid-sequence alignments of RP1L1 from 10 species reported in the NCBI database are shown. Amino acid position of 1210 is indicated by arrows. (c) Direct sequencing results of* PCDH15* gene were shown. Sequence chromatograms for normal control (top) and II-2 (bottom) are shown. II-2 had a heterozygous c.2768C>T mutation. (d) Direct sequencing results of* RPGRIP1* gene are shown. Sequence chromatograms for normal control (top) and Case II-2 (bottom) are shown. Case II-2 had a heterozygous c.3634G>A mutation. (e) Direct sequencing results of* GPR98* gene were shown. Sequence chromatograms for normal control (top) and Case II-2 (bottom) are shown. Case II-2 had a heterozygous c.7264C>T mutation.

Mutation analysis of* RP1L1* of Case II-2 identified a novel homozygous mutation (Figure 6(a), Table 2). The homozygous mutation was c.3628T>C in exon 4 which resulted in the substitution of proline for serine at amino acid position 1210. Parental consanguinity was denied by the patient. This mutation has not been reported in the single nucleotide polymorphisms (SNP) database (http://www.ncbi.nlm.nih.gov/projects/SNP/), 1,000 gene project database, Japanese 1,500 exome database, the European genome database, and Exome Variant Server (http://evs.gs.washington.edu/EVS/) or in earlier reports [3, 4, 42]. This mutation was also not present in 460 ethnically matched control alleles. The serine at position 1210 is well conserved among the RP1L1 family in other species (Figure 6(b)). We have previously reported the presence of the p.S1199C mutation of* RP1L1* in an OMD patient [2]. Amino acid residues surrounding these residues, 1193 to 1212, were well conserved among the RP1L1 family proteins (Figure 6(b)). This mutation was predicted to be probably damaging with a score of 1.00 by PolyPhen-2. The SIFT tool analysis revealed a score of 0 that predicted that the replaced amino acid is potentially damaging and would not be tolerated. PMut predicted that this mutation is pathological. Aligned GVGD predicted this mutation as Class C65 which indicates that this protein will most likely interfere with protein function.

We examined the family members and confirmed that the proband's daughter (III-2) and two grandsons (IV-1, IV-2) were heterozygous carriers of c.3628T>C (Figure 1, Figure 6(a)). A brother of the proband (II-1) was homozygous for the wild type allele of c.3628 T (Figure 1). Clinical examinations including BCVAs, slit-lamp biomicroscopy, fundus ophthalmoscopy, SD-OCT, focal ERGs, and mfERGs were performed on proband's daughter and grandsons (III-2, IV-1, and IV-2). We also measured the BCVAs, slit-lamp biomicroscopy, ophthalmoscopy, and SD-OCT of proband's brother (II-1). The findings of all examinations were normal. The OCT findings of the family (II-1, III-2, IV-1, and IV-2) are shown in Figure 3. The results of focal macular ERGs and mfERGs of proband's daughter are shown in Figure 5.

### 3.3. Molecular Genetic Analysis Using Next-Generation Sequencing (NGS)

To search for the possibility that the cone dystrophy phenotype of the patient was caused by a gene defect other than the* RP1L1* mutation, we performed NGS analysis with an exon sequencing array targeting 123 known genes associated with retinal diseases. The NGS approach identified only heterozygous missense changes in* PCDH15* (p.P923L),* RPGRIP1 *(p.V1211I), and* GPR98* (p.L2422F; Table 2). Subsequently, we designed primer pairs for PCR direct sequencing to amplify the regions identified by NGS. The results of PCR direct sequencing analysis of the family members are shown in Figures 1 and 6 (Figures 6(c)–6(e)). All three mutations identified by NGS approach did not cosegregate with the cone dystrophy phenotype, and the results of PolyPhen-2 and SIFT predicted the mutations to be benign and/or tolerated (Figure 1, Table 2). The* RP1L1* mutation was the only one that cosegregated with the cone dystrophy phenotype in the homozygous state and both simulation programs predicted it to be damaging.

### 3.4. High-Resolution Imaging Analysis

**Figure 7:**
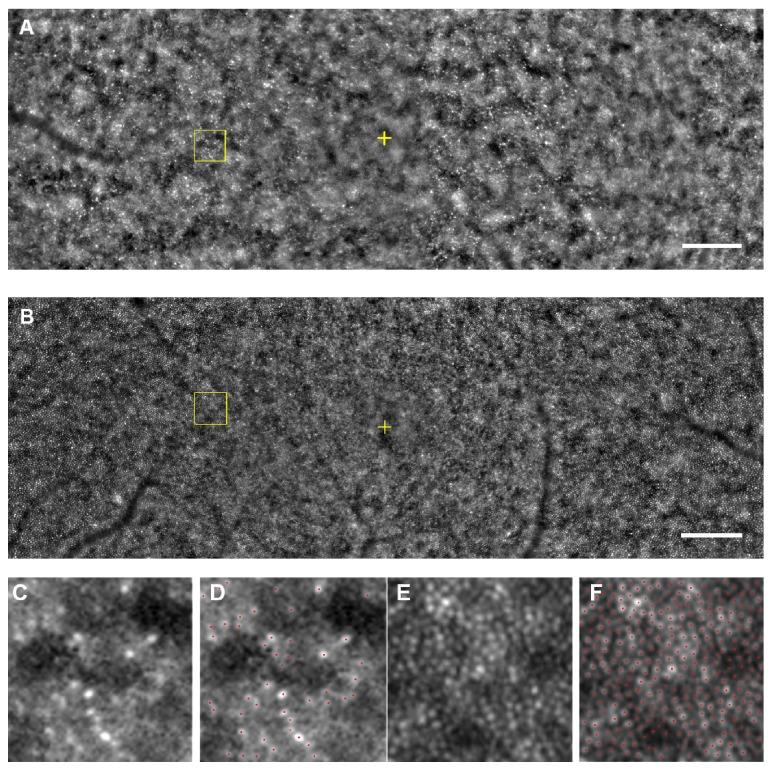
Adaptive optics images. A montage of low-magnification AO image of Case II-2 (A) and Case III-2 (B) is shown. Regular cone mosaics are not observed throughout the posterior pole in AO image of Case II-2, while AO image of Case III-2 shows well-ordered cone mosaic. Yellow cross indicates the fovea. Bar = 200 *μ*m. (C, D) Magnified view of the area outlined in (A) is shown. Regular cone mosaics are not observed in the region (C). Distance of the area from the foveal center is 600 *μ*m. A size of 100 × 100 *μ*m area is shown. (D) Cone labeling results in same region as (C) are shown. Red dots indicate estimated cones. (E, F) Magnified view of the area outlined in (B). Regular cone mosaics are observed (E). Distance of the area from the foveal center is 600 *μ*m. A size of 100 × 100 *μ*m area is shown. (F) Cone labeling results in same region as (E) are shown. Red dots indicate estimated cones.

**Figure 8:**
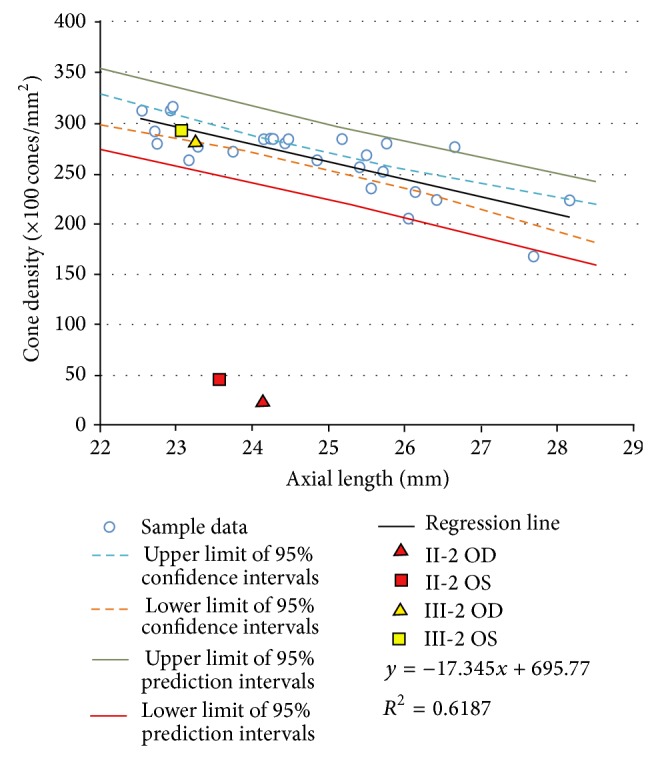
Statistical data of adaptive optics analyses. Relationship between cone density and axial length was obtained from 26 normal control samples. There was a statistically significant negative correlation between cone density and axial length (*R* ^2^ = 0.6187). Upper and lower limit of 95% confidence intervals, 95% prediction intervals, and regression line of normal controls are shown. The results of estimated cone density and axial length in Patients II-2 and III-2 are plotted as indicated marks.

High-resolution* en face* AO imaging was performed on Cases II-2 (proband) and III-2. The cone mosaic of Case II-2 was disrupted throughout the horizontal 12-degree diameter region surrounding fovea (Figures 7(A), 7(C), and 7(D)). The AO images of Case III-2, a daughter of the proband, showed a well-ordered cone mosaic (Figures 7(B), 7(E), and 7(F)). We examined the cone density at 600 *μ*m from the fovea and the axial length of 26 normal control eyes. The values of normal control group were compared to the values of Cases II-2 and III-2 (Figure 8).

A significant negative correlation between the axial length and cone density has been reported [43, 44], and it was 0.6187 for the normal controls in our study. The cone density of both eyes of Case III-2 was within the 95% prediction interval, whereas the cone densities of Case II-2 were far outside the 95% prediction interval for both eyes (Figure 8). Because the cone labeling and cone counting were performed manually for the images from Case II-2, the estimated cone density may not be an accurate number of healthy cones. However, we could recognize that the arrangement of cone mosaic was not normal throughout the investigated region of the eyes in Case II-2. By comparing the ellipsoid and interdigitation zones of the OCT images against AO images of Cases II-2 and III-2, a decreased cone density in the AO images of II-2 was consistent with the disrupted ellipsoid and interdigitation zones in the OCT images. Also, a well-ordered cone mosaic in III-2 was consistent with clearly distinguishable ellipsoid and interdigitation zones in the SD-OCT images. Because both the SD-OCT images of ellipsoid and the interdigitation zones and AO images with the focusing depth adjusted at the depth of maximum cone image intensity reflect the morphological feature of the same region, the AO and SD-OCT findings in the family were in good agreement.

## 4. Discussion

The homozygous mutation of the* RP1L1* gene in our case was a missense mutation with a substitution of proline for serine at amino acid position 1210. Heterozygous carriers of the p.S1210P mutation in her family did not have the phenotype of cone dystrophy or OMD despite the intense screening including high-resolution AO analysis. Genetic and phenotypic studies of the family members suggested that the homozygous p.S1210P mutation in* RP1L1* is able to cause cone dystrophy without affecting heterozygous individuals. Thus, our results suggest that the mutation of* RP1L1* gene can cause autosomal dominant OMD and autosomal recessive cone dystrophy.

Several genes which can cause both autosomal dominant and recessive photoreceptor degeneration have been reported. There are at least five genes,* RHO, NRL, NR2E3*,* CRX,* and* RP1,* associated with both autosomal dominant retinitis pigmentosa (ADRP) and autosomal recessive retinitis pigmentosa (ARRP) [45–48]. The reported mutations in* RHO, NRL, NR2E3,* and* CRX* are predominantly missense changes resulting in dysfunction of the mutant proteins. Therefore, these mutations most likely have dominant negative or gain-of-function effects on the molecular mechanism of RP [45–47]. In contrast, mutations in the* RP1* gene are predominantly truncation mutations resulting in premature termination codons [48]. Davidson et al. identified two unrelated RP patients with homozygous mutations in the* RP1L1* gene [3]. The mutations were a homozygous missense mutation with prediction of benign and tolerated by PolyPhen-2 and SIFT, respectively, and a homozygous premature termination mutation in* RP1L1.* These two patients with recessive RP had typical signs of RP including intraretinal bone spicule pigment deposits in the periphery and attenuated retinal vessels.

There have been approximately 1,000 nonsynonymous SNPs reported for the* RP1L1* gene with missense and frameshift variants in the SNP database. Almost all of these variants including 11 frameshifts with premature termination of the RP1L1 protein were nonpathogenic. Although none of the frameshift variants reported in the SNP database was found in the population-frequency data, most of them were reported in multiple independent studies. These findings indicate that a loss-of-function effect in* RP1L1* gene may not be pathogenic.

At present, several putative pathogenic variants of the* RP1L1* gene have been reported; however, only a variant, p.R45W, was shown to be in the multiple OMD reports [1–6]. Therefore, it is very important to evaluate new* RP1L1* variants carefully to determine whether they are pathogenic. Our case had a variation in the amino acid position 1210 which is close to the region of the reported disease-causing mutations (p.S1199C and p.G1200A) [2, 3]. The region between amino acid positions 1193 to 1212 is well conserved among species. Computational mutation analyses of all three mutations (S1199C, G1200A, and S1210P) are strongly suggested to be disease-causing, while most of the other putative* RP1L1* mutations are predicted to be benign or tolerated in at least one analysis program [2–4]. These results support the hypothesis that missense mutations in this region have a gain-of-function effect.

Our cone dystrophy case had similar characteristics as those of OMD. The fundus was normal appearing but with distinctive SD-OCT findings, for example, blurring of the ellipsoid and discontinuous interdigitation zones [2, 4, 5, 14]. Electrophysiological examinations showed severe dysfunction of the central cones with mild dysfunction of the peripheral cones. Usually, OMD patients have depressed cone sensitivity only in the macula [11, 15]. However, an OMD patient with* RP1L1* mutation (p.R45W) has been reported who had slight reductions of cone function in the full-field ERGs suggesting that the dysfunction of* RP1L1* can cause cone dysfunction detectable in the full-field ERGs [5]. Therefore, we suggest that our case had a cone dysfunction which may represent an extension of the OMD phenotype, unlike the cases reported by Davidson et al. with the typical RP phenotype.

Our study has a number of limitations. Our data do not explain why the heterozygous carriers of S1210P mutation did not have a mild cone dystrophy or OMD. One hypothesis for this is that the mutation is not pathogenically damaging enough to cause phenotypic alterations in heterozygous individual because the mutated amino acid position is peripheral to the conserved region of the gene. So far, a detailed three-dimensional conformation and the function of the RP1L1 protein have not been published, and the effects of the missense mutation on protein function have also not been published. Although we could estimate the effect of the variance of* RP1L1* gene by computational mutation analysis and their amino acid conservation in other species, we need to confirm the missense effect by their functional aspect, for example, making knock-in mice with the mutation, and protein interaction assay with mutant proteins. We performed NGS analysis to search for possible candidate genes other than the* RP1L1* that might have caused the cone dystrophy phenotype of patients. Although we did not find any possible disease causing mutations in other genes, the mutation detection rate of our NGS analysis was 57% of cases of known and novel mutations [34]. Therefore, we cannot exclude the possibility that other mutations or large deletions not detectable by the methods used in this study contributed to the phenotype.

We have demonstrated a family with a possible autosomal recessive* RP1L1* mutation, but it is important to note that we have found the mutation only in one family. Although we accumulated the evidence to possibly verify the presence of autosomal recessive cone dystrophy with* RP1L1* mutation, another patient with the same mutation will be needed to confirm the effect of the mutation.

## 5. Conclusions

In conclusion, our findings show the possibility that homozygous p.S1210P exchange in the* RP1L1* gene can cause cone dystrophy, which would then extend the phenotype of OMD.

## Figures and Tables

**Table 1:** Target genes for NGS analysis.

*ABCA4 *	*ADAM9 *	*AIPL9 *	*ARL6 *	*ASTN2 *	*ATXN7 *
*BBS1 *	*BBS10 *	*BBS12 *	*BBS2 *	*BBS4 *	*BBS5 *
*BBS7 *	*BBS9 *	*BEST1 *	*C1QTNF5 *	*C2orf71 *	*C8orf37 *
*CA4 *	*CABP4 *	*CACNA2D4 *	*CC2D2A *	*CDH23 *	*CDHR1 *
*CEP290 *	*CERKL *	*CHM *	*CLN3 *	*CLRN1 *	*CNGA1 *
*CNGB1 *	*CRB1 *	*CRX *	*CYP4V2 *	*DFNB31 *	*DHDDS *
*EYS *	*FAM161A *	*FLVCR1 *	*FSCN2 *	*GNPTG *	*GPR98 *
*GUCA1A *	*GUCA1B *	*GUCY2D *	*IDH3B *	*IMPDH1 *	*IMPG2 *
*INVS *	*IQCB1 *	*KCNJ13 *	*KLHL7 *	*LCA5 *	*LRAT *
*LZTFL1 *	*MAK *	*MERTK *	*MFRP *	*MKKS *	*MKS1 *
*MYO7A *	*NMNAT1 *	*NPHP1 *	*NPHP3 *	*NPHP4 *	*NR2E3 *
*NRL *	*OAT *	*OFD1 *	*OTX2 *	*PAF1 *	*PANK2 *
*PCDH15 *	*PDE6A *	*PDE6B *	*PDE6C *	*PDE6G *	*PDZD7 *
*PEX1 *	*PEX7 *	*PHYH *	*PITPNM3 *	*PRCD *	*PROM1 *
*PRPF3 *	*PRPF31 *	*PRPF6 *	*PRPF8 *	*PRPH2 *	*RAX2 *
*RBP3 *	*RBP4 *	*RD3 *	*RDH12 *	*RDH5 *	*RGR *
*RHO *	*RIMS1 *	*RLBP1 *	*ROM1 *	*RP1 *	*RP2 *
*RP9 *	*RPE65 *	*RPGR *	*RPGRIP1 *	*RPGRIP1L *	*SAG *
*SEMA4A *	*SNRNP200 *	*SPATA7 *	*TMEM237 *	*TOPORS *	*TRIM32 *
*TTC8 *	*TTPA *	*TULP1 *	*UNC119 *	*USH1C *	*USH1G *
*USH2A *	*WDPCP *	*ZNF513 *			

**Table 2:** Summary of RP1L1 mutation, NGS results, cosegregation, and computational prediction results.

Gene	Mutation	Cosegregation	PolyPhen-2	SIFT
*RP1L1 *	c.3628T>C p.S1210P	Yes	Probably damaging	Damaging
*PCDH15 *	c.2768C>T p.P923L	No	Benign	Damaging
*RPGRIP1 *	c.3634G>A p.V1212I	No	Probably damaging	Tolerated
*GPR98 *	c.7264C>T p.L2422F	No	Benign	Tolerated
